# Cell-Specific DNA Methylation Signatures in Asthma

**DOI:** 10.3390/genes10110932

**Published:** 2019-11-15

**Authors:** Andrée-Anne Hudon Thibeault, Catherine Laprise

**Affiliations:** 1 Département des sciences fondamentales, Université du Québec à Chicoutimi (UQAC), Saguenay, G7H 2B1 QC, Canada; andree-anne.hudon-thibeault1@uqac.ca; 2 Centre intersectoriel en santé durable (CISD), Université du Québec à Chicoutimi (UQAC), Saguenay, G7H 2B1 QC, Canada; 3 Quebec Respiratory Health Network, Quebec, G1V 4G5 QC, Canada

**Keywords:** DNA methylation, eosinophil, airway epithelial cell, monocyte, airway smooth muscle cell, lymphocyte B

## Abstract

Asthma is a complex trait, often associated with atopy. The genetic contribution has been evidenced by familial occurrence. Genome-wide association studies allowed for associating numerous genes with asthma, as well as identifying new loci that have a minor contribution to its phenotype. Considering the role of environmental exposure on asthma development, an increasing amount of literature has been published on epigenetic modifications associated with this pathology and especially on DNA methylation, in an attempt to better understand its missing heritability. These studies have been conducted in different tissues, but mainly in blood or its peripheral mononuclear cells. However, there is growing evidence that epigenetic changes that occur in one cell type cannot be directly translated into another one. In this review, we compare alterations in DNA methylation from different cells of the immune system and of the respiratory tract. The cell types in which data are obtained influences the global status of alteration of DNA methylation in asthmatic individuals compared to control (an increased or a decreased DNA methylation). Given that several genes were cell-type-specific, there is a great need for comparative studies on DNA methylation from different cells, but from the same individuals in order to better understand the role of epigenetics in asthma pathophysiology.

## 1. Introduction

According to the World Health Organization, asthma is one of the major noncommunicable diseases [1]. The highest prevalence (more than 20%) is observed in countries of Australasia, Europe, North America and in some parts of Latin America, while a lower one (5%) was in the Indian subcontinent, Asia-Pacific, Eastern Mediterranean and Northern/Eastern Europe [2]. The prevalence of asthma is increasing and is associated with environmental factors, such as urbanisation [3]. In high-income countries, asthma is mostly related to allergy predisposition (atopy) [2]. Asthmatic individuals are characterised by a loss of breath, wheezing, airway constriction, mucus production and long-term remodelling of the lung, including thickening of the basement membrane, smooth muscle proliferation and fibrosis [4]. Several inflammatory cells interact, via the production of inflammatory mediators, including mastocytes, basophils, lymphocytes, dendritic cells, eosinophils, and sometimes neutrophils to target for example epithelial cells, fibroblasts, vascular cells and airway smooth muscle cells [5].

**Figure 1:**
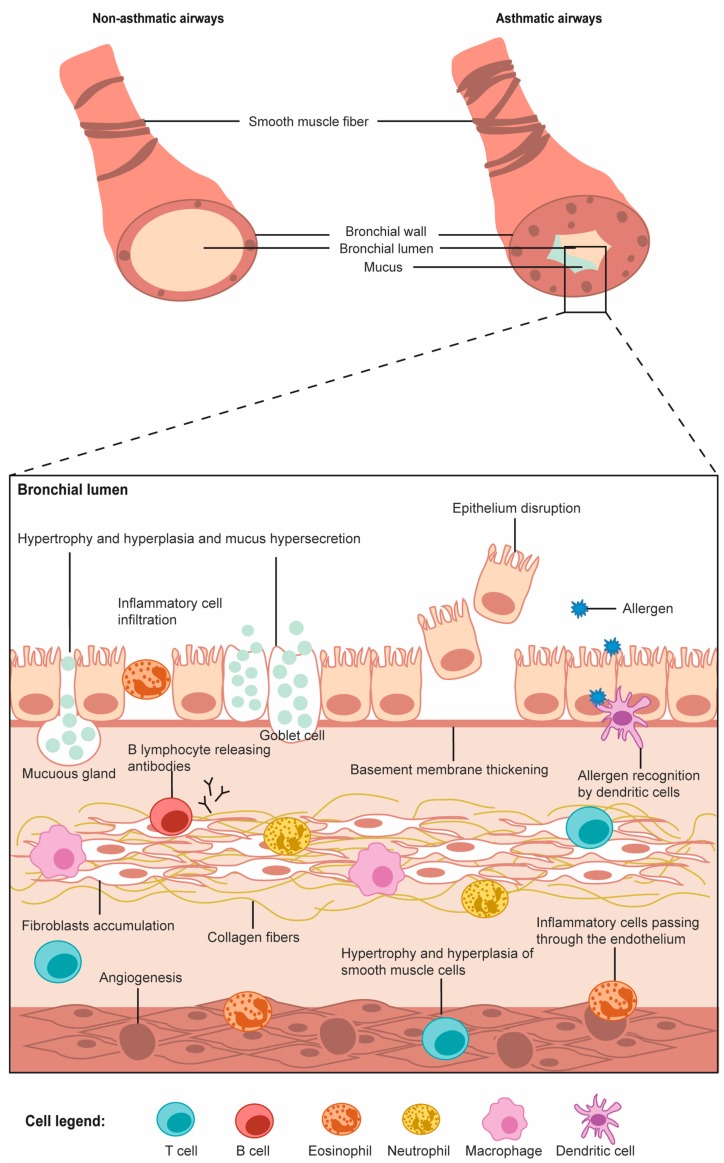
Schematic representation of immune and respiratory tract cells involved in asthma pathophysiology.

Briefly, allergens are taken up by dendritic cells and basophils, which migrate to lymph nodes where they present the antigens to T cells [4,6]. This leads notably to CD4+ T-cell differentiation into T helper 2 (Th2) population, Th17 and regulatory T cells (Tregs), which are key regulators of Th2 and Th17 cells when both of these are differentiating [7,8]. Th2 differentiation is associated with the secretion of cytokines, such as interleukin (IL)-4, IL-5 and IL-13, which are involved in B cell class switching, mastocyte activation and eosinophil recruitment [4]. Th17 cells are related to steroid insensitivity phenotype [6,9,10]. They produce IL-17 that induces IL-8, which plays a role in neutrophil inflammation and airway remodelling [6]. While less characterised, Th1 cells also have a role by secreting IFNγ, which activates macrophages that release cytokines affecting airway smooth muscle cells and leading to airway hyper-responsiveness [11,12]. Moreover, as for CD4+ T cells, CD8+ T cells also differentiate into subpopulations, such as T cytotoxic 9 (Tc9) cells [13]. These, via their cytokine secretion, rather than the typical cytotoxic mode of action involving CD8+ T cells, cooperate with Th2 cells for airway infiltration and induction of key features of asthma [13]. In the late phase of the disease, tissue eosinophilia is observed [14]. This results from the action of mastocytes and Th2-derived cytokines [14]. The processes involved in asthma pathophysiology are illustrated in Figure 1.

The eosinophils are involved in the development of characteristic features specific to asthma, such as airway remodelling and hyper-responsiveness, heightened immune response and initiation of allergic airway inflammation [14]. Eosinophilic inflammation is considered a characteristic of allergic asthma that is more common in childhood than adulthood onset [15]. The chronicity of this phase can lead to long-term remodelling of the lung [4]. Damage to airway epithelium is followed by the deposition of the extracellular matrix (ECM) [16]. Secretion of phospholipid surfactants by epithelial cells at the interface of the airway and alveoli, which prevents alveolar collapse is also altered in individuals with asthma [17,18]. The humoral response is characterised by an important secretion of IgE antibodies [4]. Binding of the allergen to IgE/FcεRI on the surface of basophils and mastocytes leads to the secretion of inflammatory mediators [4,6]. The granule contents of mastocytes also act on neurons to induce bronchoconstriction and goblet cells to mucus production [4].

The genetic contribution to asthma has been widely studied, with the identification of numerous single nucleotide polymorphisms (SNPs), as well as new loci with minor effect on the phenotype [19], but the missing heritability that is thought to arise from gene-environment interactions remains to be characterised [20]. Indeed, it is very likely that environmental exposures, which have been associated with asthma, contribute to the increased prevalence of asthma [21]. Environmental factors contributing to asthma exacerbation include, among others, the mode of delivery at birth, the use of antibiotics in childhood, the exposure to tobacco smoke and an industrialised lifestyle [21]. There are also protection factors, such as passive transfer of maternal antibodies via breastfeeding and some early infections that favour Th1 cell responses (production of anti-inflammatory cytokines) [21]. Epigenetics, recently defined by Cavalli and Heard (2019) as “*the study of molecules and mechanisms that can perpetuate alternative gene activity states in the context of the same DNA sequence*,” has been suggested to be an important contributor to several disease phenotypes [22]. Bellani (2019) illustrated the complementarity of genetics and epigenetics to allow for asthma phenotype development; the former sets up vulnerabilities to disease that can be activated by the latter [23]. Among the different types of epigenetic modifications, the most described, and on which we will focus in this review, is DNA methylation. This methylation reaction is catalysed by a family of DNA methyltransferases (DNMT1, DNMT2 and DNMT3) [24]. The contribution of DNMT2 to DNA methylation would be low, since its methyltransferase activity was reported to be weak or null, but would rather be more important on tRNAs [25,26,27,28]. Ten-eleven translocation (TET) proteins also indirectly promote DNA demethylation by oxidising 5-methylcytosine (5mC) to 5-hydroxymethylcytosine (5hmC) and further oxidisation products [29]. As a rule of thumb, DNA methylation in the promoter regions of a gene leads to transcriptional repression, by physically blocking binding of transcription factors and altering the accessibility of the machinery [24]. DNA methylation in other regions, such as in gene body may also affect the expression [30]. These regions are notably more subjected to hypermethylation in actively transcribed genes [31]. By determining which regions of the genome are accessible and expressed, DNA methylation can lead to disease predisposition [32].

Since 2016, epigenetic modifications associated with asthma have been reviewed by several authors [33,34,35], and more specifically with a focus on histone modification [36,37], translation to clinical applications approaches [38,39,40], its prediction and diagnosis [41], mucus hypersecretion [42], and prenatal maternal exposure to tobacco [43]. Most of the literature on DNA methylation in asthma was obtained from whole blood, or peripheral blood mononuclear cells (PBMCs), which is composed of a mixed population made up of lymphocytes and monocytes [44]. Deconvolution approaches have been developed in order to infer cell-type proportion from DNA methylation data from these set of samples [45]. However, using these tools do not allow for determining differential methylation in each various cell types. For this, isolated cells should be studied separately. This is especially relevant for immune cell from the blood and for cells of the respiratory tract, which play different roles in asthma pathophysiology. In this review, we compare cell-specific alteration regarding DNA methylation observed in asthmatic individuals. Genes with modifications in the DNA methylation were assigned to different categories of asthma pathophysiology (respiratory tract function, immune cell and immune functions), using the UniProt knowledgebase and the Gene Ontology for molecular function and biological process [46], and make comparisons among different cell types. A summary of the studies, techniques, cells and data sources used for the figures are found in Table S1. Studies that have assessed DNA methylation modification in an asthmatic population compared to non-asthmatic control in isolated cells (eosinophils, monocytes, lymphocytes B, airway epithelial cells or airway smooth muscle cells) were included.

Cell-specific DNA methylation modification studies allow for determining how proxies, such as whole blood or buccal samples are reliable to assess this mechanism in specific genes that could be used as biomarkers. Moreover, this type of methylation contributes to understanding the environmental impact on the complex interplay between immune and respiratory tract cells in asthma pathophysiology. More specifically, this information is relating to the asthma endophenotypes, characterised by different treatment responses and outcomes, which still lacks characterisation [47,48,49,50].

## 2. Cell-Specific DNA Methylation Modifications Associated with Asthma

DNA methylation, from whole blood cells or PBMCs in association with asthma, has been the subject of a recent review by Edris et al. (2019) [51]. It gives evidence that an important amount of data on DNA methylation was obtained using mixed cell types from peripheral or cord blood (10 out of the 16 studies included) among which only three have validated some results regarding specific cells taken individually [51]. Thousands of differentially methylated sites were identified in adults with asthma, but only 41 cytosine-phosphate-guanine (CpGs) were replicated in at least one other study, and two CpGs (associated with *DICER1* and *ABCB9*) were in three [51]. Moreover, a large overlap among atopy and asthma was observed in most studies [51]. Alterations in DNA methylation seen in whole blood are reproduced in purified eosinophils and with a larger magnitude of effect [52,53,54]. This highlights one of the benefits by studying specific cell types in order to improve the strength of the analyses. This is consistent with the methylation pattern of eosinophils, overrepresented and considered as a confounding factor, due to their variable concentration in whole blood samples [52,55,56]. This strong confounding effect appears to be driven by differences in eosinophils in males [55]. Moreover, a cluster and/or a functional analysis of different DNA methylation in whole blood have shown an “eosinophilic signature” [52,54]. As eosinophil counts and IgE are related to each other, the association of DNA methylation in whole blood with the levels of these antibodies also suggests an eosinophil signature [54,56].

In respiratory epithelial cells, only a small overlap is observed with whole blood and magnitude of changes is about 10 times larger than what was seen in the peripheral blood [52,57,58,59], suggesting specific targets in airway epithelial cells (AECs). Indeed, when comparing AEC to PBMC, a cell-specific signature consisting of 80 CpGs sites across 67 genes were differentially methylated, regardless of disease status [59]. Hence, DNA methylation from whole blood cells/PBMCs is more representative of eosinophils, but less of a component from the respiratory tract. This is the first indication that cells of the immune system and respiratory tract have different patterns of DNA methylation in asthmatic individuals.

### 2.1. Cells of the Immune System

Circulating components of the immune system include polymorphonuclear cells or granulocytes (neutrophils, eosinophils and basophils), as well as mononuclear cells (lymphocytes, monocytes, dendritic cells and NK cells) [60]. Most of these have already been isolated via immunomagnetic sorting from whole blood [52,53,61,62,63,64,65], but only a few studies have evaluated DNA methylation associated with asthma.

Different immune cell types display specific patterns of DNA methylation that are retained throughout the lineage trajectory [66,67]. For instance, myeloid lineage commitment is marked by DNA methylation depletion at binding sites of key transcription factors, such as erythroid transcription factor (*GATA1)* and T-cell acute lymphocytic leukaemia protein 1 (TAL1) [67]. Overall, DNA methylation differences are linked to cell-type-specific transcription levels observed by RNA sequencing [67]. There are also several differences between peripheral blood and any of the other sources of hematopoietic stem cells and multipotent progenitors (fetal liver, cord blood and bone marrow) [67]. Concerns that PBMC methylation differences are confounded by blood cell composition have been previously raised [68]. By comparing purified cell populations from peripheral blood, the authors conclude that, in unsorted mononuclear cells, such as PBMCs, DNA methylation is more representative of CD8+ T cells, and to a lesser extent of CD4+ T cells [68]. This is true for adult peripheral blood, but not in the one from neonatal cord [68]. This was observed in non-pathological conditions and raised questions about DNA methylation from specific cell type in the context of asthma.

#### 2.1.1. Granulocytes: Eosinophils, Neutrophils, Basophils and Mastocytes

**Figure 2:**
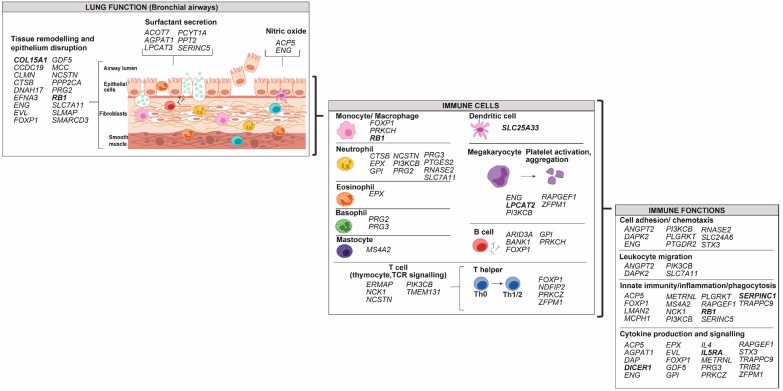
Gene targets for modification in DNA methylation from eosinophils in individuals with asthma. Genes were classified according to their potential role in lung function, in immune cells and in immune functions using the UniProt knowledgebase and Gene Ontology for molecular function and biological process [46]. Decreased methylation in asthma versus control was observed in all genes represented in the figure. Bold: Genes that were replicated in another study. *NTRK1*, *NCF2* and *ZFPM1* not included because the opposite effect on DNA methylation was reported [53,71,72].

DNA methylation from granulocytes has been mainly studied in eosinophils. Figure 2 shows genes with alteration in DNA methylation from purified eosinophils and associated with asthma pathophysiology taken from three studies (Tables S1 and S2) [52,53,54]. These data were all obtained from samples of the same cohort (The Saguenay‒Lac-Saint-Jean asthma familial one) from our laboratory [69]. All gene targets display a decreased methylation in asthmatic individuals and are diversified among this disease pathophysiology and immune system components and functions. Interestingly, a potential transcription factor in eosinophil lineage-active binds to an enhancer-like region within the *IL5RA* promoter has been identified [70], and alteration in DNA methylation of this gene was observed in asthmatic individuals (Figure 2).

Few data are available on DNA methylation from other granulocytes. It was, however, observed that neutrophils have a specific combination of epigenetic marks (histone modifications and DNA methylation), when compared to monocytes [73], which suggest that they could be differently affected in asthmatic individuals, when compared to monocytes. Moreover, in human mast and basophil cell lines, hypomethylation of the promoter regions of histidine decarboxylase (*HDC*), which catalyses histamine formation, was associated with an increase of its expression [74]. This regulation of *HDC* was specific to these cells as compared to other cell lines (human cervical cancer HeLa and K562 erythroleukemia cells) [74]. Here again, this emphasises the interest of studying specifically these cells, especially for DNA methylation in *HDC*, which is involved in the synthesis of a crucial inflammatory mediator associated with allergic asthma [75] that might not be found in other immune cell types or respiratory tract cells.

#### 2.1.2. Monocytes and Macrophages

**Figure 3:**
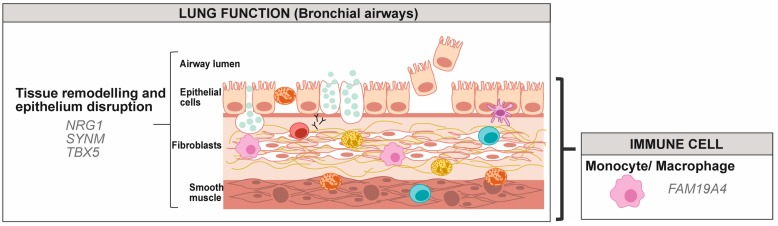
Gene targets for modification in DNA methylation from monocytes in individuals with asthma. Genes were classified according to their potential role in lung function, in immune cells and in immune functions using the UniProt knowledgebase and Gene Ontology for molecular function and biological process [46]. Increased methylation in asthma versus control was observed for all genes represented in the figure.

In individuals with asthma, aberrant differentiation of monocytes is observed, notably characterised by a predominance of CD14+/CD16+ cells in blood, typical of tissue macrophages [76,77]. DNA methylation differs between the subsets of monocyte [78], and its localised remodelling is observed during monocyte differentiation into classically activated (M1) and alternatively activated (M2) macrophages [79,80,81]. More specifically, during monocyte to macrophage differentiation, a specific phagocytic gene network is demethylated by TET enzymes [82]. Consistent with this, decreased methylation generated by DNMT3b knockdown or inhibitors of DNMT is associated with the promotion of macrophage differentiation [80]. Only one study has assessed DNA methylation in monocytes in asthmatic individuals, comparing eosinophilic asthma (EA), paucigranulocytic asthma (PGA) or neutrophilic asthma (NA) [63]. While EA and NA individuals are characterised by infiltration of eosinophils and neutrophils, respectively, into the airways, PGA subjects have normal levels of both, but elevated alveolar macrophages. In monocytes from EA, PGA and NA, 413, 495 and 89 loci showed altered DNA methylation, respectively, but only nine sites were common to all three types of asthma, all hypermethylated [63]. The ones relevant to asthma pathophysiology are presented in Figure 3. Genes targeted by an alteration in DNA methylation would be restricted to tissue remodelling and epithelium disruption, as well as to macrophage function. Macrophages are derived from recruited circulating monocytes and their importance in lung tissue homoeostasis and pathological remodelling in diseases, such as asthma was recently reviewed [83], consistent with what is depicted in Figure 3 and Table S3.

The pathway analysis of asthma subtypes has identified specific clusters associated with EA (purine metabolism, calcium signalling and ECM-receptor interaction), PGA (neuroactive ligand-receptor interaction and ubiquitin-mediated proteolysis) and NA (a Wnt signalling pathway, involved in the early development of the airway smooth muscle) [63]. This also highlights the importance of having the subtype of asthma well characterised. Moreover, this single study on DNA methylation from monocytes related to asthma still lack replication, but demonstrates the feasibility of such studies.

#### 2.1.3. Dendritic Cells

Dendritic cells are professional antigen presenters and key players in initiating the immune response in asthma [84]. Dendritic cell development and maturation are associated with a loss of DNA methylation across many regions involved in their lineage specification and response to immune stimuli [85,86]. Changes in dendritic cell markers are related to the clinical efficacy of allergen immunotherapy [6], and manipulations of such functions have been suggested for a novel asthma treatment [87]. No data is currently available on DNA methylation from human dendritic cells associated with asthma, but animal models can give some insights on the importance of studying this cell type, as changes in their DNA methylation were related to transgenerationally transmitted asthma susceptibility from mothers with allergies [88] and with prenatal exposure to diesel exhaust particles or concentrated urban air particles [89]. In the latter study, they observed altered loci shared across three generations that were not linked to known allergies/asthma genes, but rather to chromatin modification. This suggests interaction with other epigenetic mechanisms, such as histone modification [89]. Hence, in the field of DNA methylation from dendritic cells, much work still has to be done, and animal studies indicate a role in the transgenerational transmission of asthma risk.

#### 2.1.4. Lymphocytes T (CD4+ and CD8+)

CD4+ T cell differentiation, polarisation and plasticity involve several epigenetic mechanisms, including DNA methylation [90]. Differentiation of naïve CD4+ T cells into Th1 cells is associated with methylation of the promoter region of IL-4 and demethylation of the interferon gamma (*IFNG*) gene [5,91]. Differentiation into Th2 cells is related to demethylation in IL-4 and IL-13 promoter regions allowing binding of transcription factors STAT6 and GATA3 [5,91]. 5-hydroxymethylcystosine (5-hmc) enrichment in the body of highly expressed genes was also observed at different stages of T cell development [92]. Regulatory T (Treg) cells are another subset with immune suppressive properties that also promote tolerance to self-antigens. Demethylation of the regulatory forkhead box protein 3 (FOXP3) is well known to be required for activation and maintaining the suppressive properties of Treg cells [93,94,95,96,97]. More specifically, the recombinant human protein phospholipase D2 (rhPLD2), which is involved in proliferation, chemotaxis and migration of lymphocytes, allows the expression of FOXP3 in CD4+CD25+Foxp3+Treg cells, which is due to demethylation of a specific region (TSDR) of FOXP3 [98]. In asthmatic individuals, increased methylation of FOXP3 in peripheral blood Treg cells has been associated with their functional impairment in an environment of high annual ambient air pollution levels compared to asthmatic individuals with low air pollution levels [99]. The differentiation of CD8+ T cells (from a naïve to an effector cell) is related to differentially methylated regions [100]. In turn, epigenetic repression of naïve-associated genes in effector CD8+ T cells can be reversed, which, in combination with demethylation of loci classically associated with effector molecules, give rise to memory CD8+ T cells [101]. While several epigenetic modifications were related to T cell differentiation in non-pathological conditions, DNA methylation from naïve cells is a crucial work that remains to be done in the field of asthma epigenetics, and that is central in order to understand the disease effect on the complex differentiation of this immune cell type.

#### 2.1.5. Lymphocytes B (CD19+)

**Figure 4:**
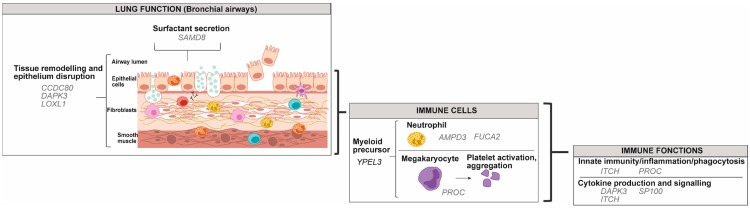
Gene targets for modification in DNA methylation from lymphocyte B in individuals with asthma. Genes were classified according to their potential role in lung function, in immune cells and in immune functions using the UniProt knowledgebase and Gene Ontology for molecular function and biological process [46]. Black: Genes with decreased methylation in asthma versus control; Gray: Genes with increased methylation in asthma versus control.

B cells that are producing IgE play a crucial role in allergic inflammation associated with asthma [5]. B cell development is influenced by DNA methylation and demethylation by DNMT1 and TET enzymes [29,102]. Differentiation from a mature naïve B cell to a germinal centre cell is combined with demethylation of several genes, with concordant inverse changes in gene expression, which involves activation-induced cytidine deaminase (AID) [102,103]. DNA methylation patterns in B cells were more concordant between control and aspirin-exacerbated respiratory disease than between allergic asthmatic individuals compared to control [104]. It is likely that lymphocytes B from allergic asthmatic individuals include a more substantial subset of IgE producing B cells, which could explain the important changes in DNA methylation within this group [104]. There is a general trend towards decreased methylation in allergic asthmatic compared to non-asthmatic individuals, but when focusing on the most differentially methylated genes that are associated with asthma pathophysiology, we observed an increase in methylation in most of these genes [104] (Figure 4 and Table S4).

#### 2.1.6. Natural Killer Cells (NK)

The impairment of natural killer (NK) cell activity has been seen in individuals with asthma, but the trend of the observed effects (their increased or decreased cytotoxicity) remains controversial [105,106,107]. NK cell activities go beyond their known role in the natural host defence against infectious pathogens. They also influence the T-cell response, interact with dendritic cells to induce their maturation and produce type 2 cytokines [107,108]. In vitro activation of NK is associated with altered DNA methylation, which reveals similarities with activated T cells, but also shows cell-specific alterations when compared with methylated T and B cells [64]. Class E basic helix-loop-helix protein 40 (BHLHE40) demethylation was notably suggested as a biomarker of NK cell activation [64]. The DNA methylation modifications of NK associated with asthma remains uncharacterised.

#### 2.1.7. Platelets and Megakaryocytes

Platelets, derived from megakaryocyte fragmentation, are traditionally associated with coagulation [109]. Their activation has also been related to asthma and a recent review demonstrates their role in terms of immunity, by interacting with immune and endothelial cells, but also by secreting immune mediators [110]. Hence, this allows platelets to play a role in lung remodelling, as well as allergic sensitisation [110]. Megakaryocyte maturation involves endomitotic replication and an exponential increase in cell ploidy [67]. A small number of genes were differentially methylated with consistent and progressive changes during megakaryocyte maturation [67]. As for the role of platelets in asthma pathophysiology, that of DNA methylation in megakaryocytes and their functions in the context of asthma remains largely uncharacterised.

### 2.2. Cells of the Respiratory Tract

In individuals with asthma, the respiratory tract is a target of the immune system, as well as an effector of this disease symptoms [111]. Different layers cover the respiratory tract (epithelial cells, goblet cells, fibroblasts and smooth muscle cells) [112], but for technical reasons, having access to these samples can be much more invasive than for blood cells. Airway epithelial cells, especially nasal cells, are more easily accessible and have been widely studied. Airway smooth muscle cells have also been investigated, but to a much lesser extent.

#### 2.2.1. Airway Epithelial Cells

**Figure 5:**
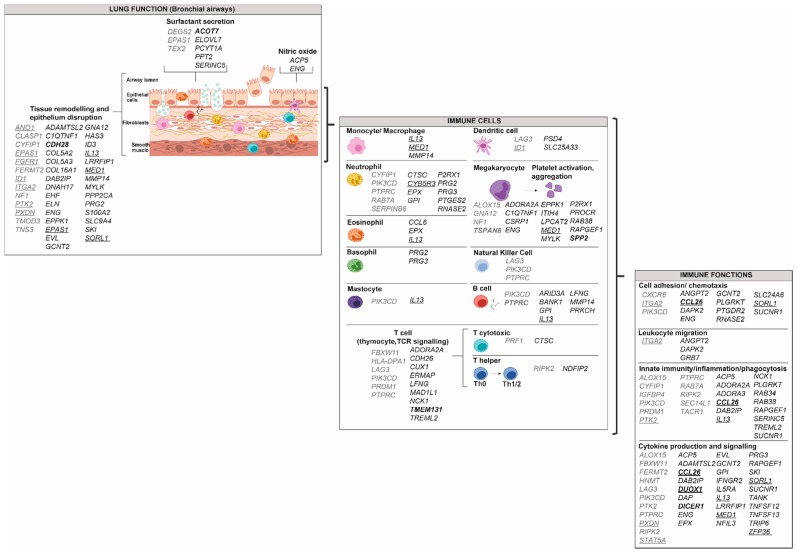
Gene targets for modification in DNA methylation from airway epithelium cells in individuals with asthma. Genes were classified according to their potential role in lung function, in immune cells and in immune functions using the UniProt knowledgebase and Gene Ontology for molecular function and biological process [46]. Gray: Genes with increased methylation in asthma versus control; Black: Genes with decreased methylation in asthma versus control; Bold: Genes that were replicated in another study, Underlined: Data obtained from bronchial epithelial cells.

Due to their localisation, nasal epithelial cells are more prone to changes following environmental exposures (direct exposition to triggers). Indeed, while the nasal epithelium was shown to be a good proxy for the airway epithelium, more DNA methylation changes were associated with asthma in nasal epithelial cells than in airway epithelia [57], suggesting a greater number of environmental insults. Epithelial cells from deeper airways to nasal epithelium were grouped in Figure 5 under the term airway epithelial cells (AECs). When comparing nasal epithelial cells to deeper AEC, we observed that *CCL26*, which was replicated in two studies, was only altered in bronchial cells, while *DUOX1* was common to the two subtypes (Table S5). Genes with increased and decreased DNA methylation are distributed among the different features of asthma pathophysiology (Figure 5). Only data from DNA methylation modifications in individuals living with asthma versus non-asthma were included [52,53,59,71,113,114] (Tables S1 and S5).

Some studies have more specifically investigated DNA methylation affected by IL-13 in AECs [114,115]. IL-13 is a member of the IL-1 family activating IL-33-specific receptor (IL1R1) and leads to upregulation of Th2-driven inflammation. It is known to be persistently elevated in the asthmatic airways [115]. An *in vitro* experiment of AECs treated with IL-13 showed that a significant proportion (2020 of 6522 CpGs) of this epigenetic signature was validated in cells isolated from asthmatic individuals [115]. Moreover, it was shown that, in AEC, a haplotype within promoter *IL33* might interact with DNA methylation to modulate asthma risk [114]. As illustrated in Figure 5, DNA methylation in promoter regions of *IL33* and *IL1R1* in asthma from bronchial epithelial cells is decreased [114]. The relevance of IL-13 in asthma pathophysiology via DNA methylation has also been evidenced in a transgenic mouse model where expression of *IL13* is induced [116]. In this model, alterations in DNA methylation were observed in genes involved in tissue remodelling, leukocyte influx and Th2 response [116]. IL-4 is also a contributor to Th2-type response and treatment of primary AECs with this cytokine resulted in demethylation of pendrin (*SLC26A4*), an anion transporter exchanging chloride for iodide or bicarbonate increased in the lung bronchial epithelium of asthmatic individuals [117]. Pendrin is involved in the regulation of the airway surface liquid and mucus production [118]. Baccarelli et al. (2012) have also observed that inducible nitric oxide synthase (*NOS2)* and IL-6 (*IL6)* have a decreased methylation associated with an increased fractional exhaled nitric oxide, a measure of lower airway inflammation in nasal cell DNA from asthmatic children [119]. These genes were not altered in asthmatic versus non-asthmatic individuals (Figure 5) and would rather be associated with asthma severity. These studies show the importance of ILs in the regulation of DNA methylation from AECs in asthma.

DNA methylation in nasal epithelium has also proven to be successful in predicting good versus poor responders to corticosteroid treatment. The methylation of pantethieianase (*VNN1*) following corticosteroid treatment is associated with good responders to treatment [120], while homebox protein OTX2 (*OTX2)* and L-lactate dehydrogenase C chain (*LDHC)* are demethylated in good responders as compared to poor responders [121]. This suggests that response to treatment involves alteration in DNA methylation in different genes than the ones that are altered in asthma.

#### 2.2.2. Airway Smooth Muscle Cells

**Figure 6:**
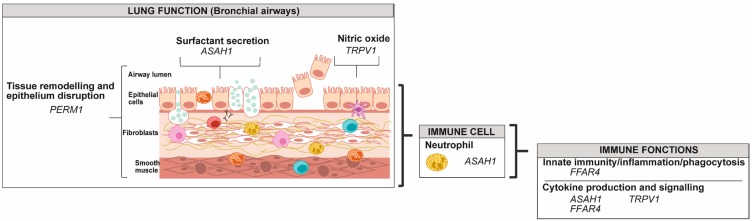
Gene targets for modification in DNA methylation from airway smooth muscle cells in individuals with asthma. Genes were classified according to their potential role in lung function, in immune cells and in immune functions using the UniProt knowledgebase and Gene Ontology for molecular function and biological process [46].

In individuals with asthma, airway smooth muscle cells (ASMCs) undergo hyperplasia [122], and 12,383 differentially methylated positions (DMPs) defined by 20% mean methylation difference, were identified when compared to controls [123]. A total of 15 DMP hub sites (central sites within a module determined by weighted gene co-expression analysis) were associated with asthma, but data on the trend of the methylation were not indicated [123]. Among the genes with DNA modification that we illustrated in asthma physiology, none were common with eosinophils, lymphocytes B, monocytes or AECs (Figure 6, Tables S6 and S7). These data remain to be validated in other studies.

## 3. Differences in DNA Methylation Associated with Asthma among Various Cell Types

Identifying gene targets for DNA methylation according to different cell types allowed for highlighting that, while in AEC and ASMC the status of the methylation (increased or decreased) is diversified; eosinophils only show genes with decreased methylation associated with asthma. Based on the general assumption that DNA methylation in the promoter region of genes is associated with their repressed expression, and that most data were obtained with the Illumina 450K array (Table S1) which is biased towards regions having high CpG content, such as promoters [124], observations presented in Figure 2 suggest that asthma leads to global increase in gene expression in eosinophils, but this affirmation still lacks empirical evidence. This is consistent with the characteristic increase in eosinophils in the airway of asthmatic individuals. Among the circulating eosinophils, different activation states are observed, characterised by their surface phenotype consisting of numerous cell surface proteins [125]. The high expression of the cell surface protein is achieved on eosinophils from bronchoaleveolar lavages, but is less often observed on circulating ones [125]. In asthmatic or allergic individuals, blood eosinophils have a greater degree of adhesion, transendothelial migration or responsiveness to chemoattractants [125]. The permissive expression of genes allowed by decreased DNA methylation could be associated with these altered functions of eosinophils in asthmatic individuals.

Opposite to what is observed in eosinophils, for several asthma features in AEC depicted in Figure 5, genes have either increased or decreased methylation. For example, many are associated with neutrophil degranulation, some having increased (*CYF1P1, PTPRC, RAB7A* and *SERPINB6*), and others decreased methylation (*CTSC, CYB5R3, EPX, GPI, P2RX1, PRG2, PRG3, PTGES2, RNASE2*). The impact of the increased and decreased methylation of different genes related to neutrophil degranulation is not clear, and further expression analysis and functional experiments would help to understanding this.

Interestingly, in monocytes, genes are rather subjected to increased methylation in individuals with asthma. Monocytes are recruited from the bloodstream and promote acute inflammation, while resident alveolar macrophages play a suppressive role in an attempt to restore homoeostasis [126]. The observation that asthma is associated with increased DNA methylation suggests an inhibitory effect on differentiation into macrophages (Section 2.1.2) and impairment in their role of the restoration of homoeostasis [126].

Both AECs and eosinophils show target genes in several aspects of asthma pathophysiology (120 and 57, respectively), among which 34 are common between the two cell types (Table S7). However, the number of genes identified is likely influenced by the number of studies, greater for AECs, and we cannot exclude their eosinophil contamination that could influence the DNA methylation portrait, at least in part. Some genes that had alteration in DNA methylation replicated in more than one study were specific with a cell type. In AECs, several genes are associated with megakaryocyte and platelet function (*DUOX1*, *SSP2*, *TSPAN8).* This is consistent with the suggested role of platelets in lung regeneration and inappropriate airway remodelling, after an injury during allergic sensitisation and inflammation [110]. In eosinophils, genes are related to tissue remodelling (*COL15A1* and *RB1*), innate immunity/inflammation/phagocytosis (*RB1* and *SERPINC1*), as well as components of the innate immunity, such as monocytes/macrophages (*RB1*), dendritic cells (*SLC25A33*), suggesting a regulatory role of eosinophils in innate immunity and associated cells. Indeed, eosinophils actively regulate a variety of immune functions through their granule products and cytokines. They notably contribute to macrophage activation and closely interact with mastocytes for their activation [127]. Some of these roles could be mediated by alteration in DNA methylation of specific genes.

If we compare the asthma features depicted for the different cell types (Figure 2, Figure 3, Figure 4, Figure 5 and Figure 6), it is especially interesting to note that genes that are involved in natural killer cells, as well as cytotoxic CD8+ T lymphocytes, are only affected in AECs. This accentuates the specific targets depending on the cell type. This is relevant to the known interactions of these cells with airway epithelial cells. For example, AECs express NK cell receptor ligands [128], and CD8+ Tc are involved in airway remodelling in a rodent model of asthma [129].

Whole blood studies of the alteration in DNA methylation, reviewed by Edris et al. (2019) were shown to be a correct predictor for some of the genes altered in eosinophils and AECs (Table S8), among which a great proportion of these are involved in granulocyte functions, notably neutrophils (*EPX*, *PRG2*, *PTGES2* and *RNASE2*), and cytokine production and signalling (*DICER1*, *EPX*, *EVL*, *IL13*, *IL5RA*, *RAPGEF1* and *ZFPM1*). Most of these genes are also common between eosinophils and AECs (Table S7). This is of great importance because it underlines the relevance of whole blood as a good proxy for specific cell functions. The cell isolation procedure adds complexity to the studies, often leading to a modest sample size included in these studies (Table S1). Hence, increasing the sample size is a crucial step in deciphering cell-specific DNA methylation alteration associated with asthma. Moreover, buccal cells were also used as proxies for airway cells. Some genes that show altered DNA methylation in buccal cell samples in asthmatic individuals or associated with asthma traits (increased fractional exhaled nitric oxide) (*ARG2*, *INAR2* and *NOS2*) are relevant to what is observed in AECs and notably the alteration of NO secretion in Figure 6 [130,131,132]. Consistent with this, a comparative study has found that nasal epithelium cells are the most representative of AECs regarding methylation profile in asthmatic individuals, while buccal cells were only moderately similar as blood DNA methylation poorly reflected the DNA methylation in AECs [133].

Cytokines are key players of the immune response in asthma pathophysiology and are the subject of numerous studies [111,134,135,136,137]. The genes targeted by changes of DNA methylation are different depending on the cell type (Table 1). Several genes associated with cytokines IL-4 and IL-5, known to be involved in asthma pathophysiology [111,137,138], are both affected in AECs and eosinophils. Some others are specifically affected by differential DNA methylation from specific cell types: IL-6, IL-7, IL-15, IL-16 and IL-18 in AECs, as well as IL-9, IL-14, IL-21 and IL-33 in eosinophils. Of interest, IL-7 and INF are known for being produced and to target AECs, respectively, and are altered in asthmatic individuals [134]. IL-18 also plays a role in CD4+ T-cell differentiation into Th1 and Th2 [135]. As for eosinophils, IL-9 is involved in goblet cell metaplasia and promotion of their accumulation [111,136], while IL-33 is an epithelial-derived cytokine [111,139]. Hence, eosinophils could contribute to altering epithelial IL-33 function.

### 3.1. DNA Methylation Machinery

The fact that, in some cell components, genes from asthmatic individuals have both increased and decreased methylation compared to a global decrease in eosinophils, leads to the question concerning the disease impact on DNA methylation machinery depending on the tissues and cell types. Factors that influence disease-associated alteration in DNA methylation include changes in DNA methyltransferases, in ten-eleven translocation (TET) proteins, as well as selective recruitment of DNA-modifying enzymes by transcription factors [31]. DNMT expression varies depending on the tissues [140], but the question of the methylation machinery being expressed in specific cells remains, since those are composed of a mixed cell population. Interestingly, DNMT3 expression can be induced by T cell receptor (TCR) signalling, which would suggest that T cells be more prone to increased methylation following TCR activation [141]. The explanation of the cell-specific DNA methylation pattern associated with asthma could reside in the DNMT-including complexes that are recruited on specific DNA sequences, rather than on the unspecific DNA methylation machinery [142]. Transcription factors involved in the recruitment of DNA modification enzymes could be altered in asthmatic individuals, depending on the cell type. For instance, p53 and methyl-CpG binding domain protein 2 (MBD2) are both known to be involved in the specific recruitment of DNMTs [142], and are both increased in asthmatic individual, in bronchial smooth muscle [143,144] and CD4+ T cells [145,146], respectively.

### 3.2. Comparative Studies

Comparative studies among different tissues or cell types have found that only a few genes have common hypermethylation or hypomethylation [147,148]. Based on the comparison of reference epigenetic profile from different cell types, tools to interpret DNA methylation data obtained from blood in the context of another tissue were developed [149]. This allows for determining informative CpGs in a surrogate tissue that can be reliably extrapolated in another one [149]. Comparative studies that use samples from the same population are necessary in order to decipher the vulnerabilities of specific cell types to alteration in DNA methylation. Such study has already been performed for multiple sclerosis, a chronic inflammatory disease, where DNA methylation was measured and compared in case of CD4+ and CD8+ T cells, CD14+ monocytes, as well as CD19+ B cells [150]. Most DMPs were observed in B cells, as compared to T cells [151]. In different subtypes of immune cells isolated from the blood (including CD4+, CD8+, CD56+, CD19+ and CD14+ cells), DNA methylation in the *GSDMB/ORMDL3* locus also showed differences between asthmatic as compared to control individuals [150]. More specifically, there is a decrease in DNA methylation in the 5’UTR of *ORMDL3* in CD8+ T cells, which might explain the increased mRNA expression in these cells compared to other blood leukocytes [150]. Hence, this cell type could be important for the connection between *ORMDL3* and asthma susceptibility [150]. Consistent with this cell-specific observation, our group has also recently identified among the 17q12-21 locus, a specific methylation block in CD4+ T cells, 3.2 kb upstream of *IKZF3* transcription start site (TSS) [65]. Multiomic approach also showed that asthma-associated SNPs modulate gene expression counts and DNA methylation levels of two CpGs within the 1.5 kb region from TSS of *GSDMA* in CD4+ T cells, but not in eosinophils [65].

Another important aspect to consider in the cell-specific epigenetic modification is the localisation of the alteration in DNA methylation, which can be observed in different regions, including promoters, actively transcribed gene bodies or intergenic regions [31]. Depletion of DNA methylation in promoter regions and the enrichment in transcribed regions are both more pronounced in highly expressed genes [147,152]. Most of the data discussed in this review were obtained using the Illumina 450K array (Table S1), which is biased towards regions with high CpG content, such as promoters [124]. Hence, this would suggest a strong probability of the inverse relationship between CpG methylation and expression. However, data on methylation elsewhere on the genes are still lacking. This requires specific techniques, such as methylC-capture sequencing that allows for assessing tissue-specific and disease-relevant regions, such as enhancers [124]. Hence, there is still a great proportion of black boxes of DNA methylation associated with asthma that remains to be discovered.

Overall, several genes with an alteration in DNA methylation are specific to the cell types in which the data were obtained. Validation of most of these observations in replication study remains to be done, and in order to confirm the relevance of these genes in asthma pathophysiology, their expression and that of protein should be further explored. Moreover, single-cell analysis is a promising complementary approach to decipher asthma pathophysiology at a micro level [153]. DNA sequencing and methylation techniques for single-cell characterisation are currently being improved and could contribute to understanding its heterogeneity among a population of one cell type [154,155].

## 4. Conclusions

Asthma has a complex pathophysiology that sometimes includes atopy features. It is both under genetic and environmental control. The latter is known to affect DNA methylation and have been the subject of several studies. EWAS have been growing in importance, submerging researchers in the field of asthma with genes as targets for alteration in DNA methylation in different cell models. In this review, we have focused on DNA methylation in specific cells of the immune system and of the respiratory tract for which data were available. Placing genes with altered DNA methylation into asthma pathophysiology allowed for identifying some features of this disease that are affected in specific cell types. Moreover, there is a great need for comparative studies on DNA methylation profile in those different cell models.

## Figures and Tables

**Table 1:** Genes with altered DNA methylation from airway epithelial cells (AECs) and eosinophils according to their effect on the different cytokines production and signalling.

Cytokines	AECs	Eosinophils
IL-1	*ACP5, CCL26, DAB2IP, FBXW11, HNMT, PXDN, RIPK2, TRIP6, SUCNR1*	*ACP5, FOXP1*
IL-2	*ACP5, HNMT, LAG3, PTPRC, RIPK2, STAT5A, ZFP36*	*ACP5*
IL-3	*NFIL3*	*PRKCZ*
IL-4	*EPX, NFIL3*	*EPX, IL4, PRKCZ, ZFPM1*
IL-5	*EPX, IL5RA*	*EPX, IL1RL1, IL5RA, PRKCZ*
IL-6	*SORL1*	
IL-7	*STAT5A*	
IL-8	*PRG3, PTPRC, TNFSF12*	*CAMP, PLA2G1B, PRG3*
IL-9	*STAT5A*	
IL-10	*EPX*	*EPX, PRKCZ, TRIB2*
IL-12	*RIPK2*	*FOXP1*
IL-13	*ALOX15, IL13*	*IL4*
IL-14		*METRNL*
IL-15	*STAT5A*	
IL-16	*RIPK2*	
IL-18	*RIPK2*	
IL-21		*FOXP1*
IL-33		*IL1RL1*
Interferon (INF)	*EVL, IFNGR2, LRRFIP1, MED1, TANK*	*EVL*
Transforming growth factor (TGF)	*ADAMTSL2, FERMT2, GCNT2, IL13, PTK2, SKI*	*ENG, GDF5*
Tumor necrosis factor (TNF)	*CCL26, DICER1, MAP3K4, PTPRC, ZFP36*	*DICER1, FOXP1*

## Supplementary Materials

The following are available online at https://www.mdpi.com/2073-4425/10/11/932/s1, Table S1: List of studies included in the cell-specific DNA methylation alteration figures, Table S2: Genes with DNA methylation alteration in eosinophils from asthmatic individuals, Table S3: Genes with DNA methylation alteration in monocytes from asthmatic individuals, Table S4: Genes with DNA methylation alteration in lymphocytes B from asthmatic individuals, Table S5: Genes with DNA methylation alteration in airway epithelial cells from asthmatic individuals, Table S6: Genes with DNA methylation alteration in airway smooth muscle cells from asthmatic individuals, Table S7: Genes with DNA methylation alteration that are common between cell types, Table S8: Genes with DNA methylation alteration that are common with blood DNA methylation alteration.
